# The Antidepressant Drug Amitriptyline Affects Human SH-SY5Y Neuroblastoma Cell Proliferation and Modulates Autophagy

**DOI:** 10.3390/ijms251910415

**Published:** 2024-09-27

**Authors:** Annagrazia Adornetto, Maria Luisa Laganà, Andrea Satriano, Ester Licastro, Maria Tiziana Corasaniti, Giacinto Bagetta, Rossella Russo

**Affiliations:** 1 Preclinical and Translational Pharmacology, Department of Pharmacy, Health and Nutritional Sciences, University of Calabria, 87036 Rende, Italy; annagrazia.adornetto@unical.it (A.A.); giacinto.bagetta@unical.it (G.B.); 2 School of Hospital Pharmacy, Department of Health Sciences, University “Magna Graecia” of Catanzaro, 88100 Catanzaro, Italy; mtcorasa@unicz.it

**Keywords:** amitriptyline, antidepressant, autophagy, neuroblastoma, cell proliferation, cytotoxicity, apoptosis

## Abstract

Amitriptyline is a tricyclic antidepressant commonly used for depressive disorders and is prescribed off-label for several neurological conditions like neuropathic pain, migraines and anxiety. Besides their action on the reuptake of monoaminergic neurotransmitters, tricyclic antidepressants interact with several additional targets that may contribute to either therapeutic or adverse effects. Here, we investigated the effects of amitriptyline on proliferation and autophagy (i.e., an evolutionarily conserved catabolic pathway responsible for the degradation and recycling of cytoplasmic material) in human SH-SY5Y neuroblastoma cell cultures. The dose and time-dependent upregulation of the autophagy marker LC3II and the autophagy receptor p62, with the accumulation of LAMP1 positive compartments, were observed in SH-SY5Y cells exposed to the amitriptyline. These effects were accompanied by reduced cell viability and decreased clonogenic capacity, without a significant induction of apoptosis. Decrease viability and clonogenic activity were still observed in autophagy deficient Atg5^−/−^ MEF and following pre-treatment of SH-SY5Y culture with the autophagy inhibitor chloroquine, suggesting that they were independent from autophagy modulation. Our findings demonstrate that amitriptyline acts on pathways crucial for cell and tissue homeostasis (i.e., autophagy and proliferation) and pose the basis for further studies on the potential therapeutic application of amitriptyline, as well as the consequences of its use for long-term treatments.

## 1. Introduction

Affecting 121 million people worldwide, depressive disorders are the second cause of Years Lived with Disability (DALY) in the age category 15–44 and are the fourth leading contributor to the global burden of disease in developing countries [1]. Depression is often a common co-morbidity in patients with chronic medical diseases including neurodegenerative diseases, strokes, epilepsy, multiple sclerosis, autoimmunity [2,3,4,5] and cancer [6,7], and it is a post-partum complication [8,9,10,11]. 

Antidepressants are the first-line medications for moderate and severe depression and are among the most widely prescribed drugs [12]. Their consumption has significantly risen in the last two decades, with a five-fold increase in the United States and a three-fold increase in European countries [13]. A further rise is linked to the recent coronavirus 2019 (COVID-19) pandemic which contributed to a worsening mental health crisis, especially among adolescents and young adults [14,15]. Aside from their use in managing depressive symptoms, antidepressant drugs are used to treat people suffering from other mental health issues and physical conditions, including anxiety, psychotic disorders, attention deficit, insomnia, migraines, neuropathic pain, premenstrual dysphoric disorder and gastrointestinal and genitourinary pathologies [16,17,18,19,20,21,22,23,24]. Moreover, following the concept of drug repurposing, antidepressant drugs are now being explored for their usefulness in diseases beyond their therapeutic indication, for example in cancer treatment [25,26].

Indeed, although the primary action of antidepressants is the regulation of monoamine concentration in the synaptic space, several studies suggest that they possess anticancer properties [25,26,27], which encompass apoptosis induction [28], the restriction of cellular energy metabolism [29], antioxidant activities [30], the inhibition of angiogenesis [31] and the modulation of the immune response [32]. 

Tricyclic antidepressants (TCAs), introduced in the market in 1959 for the treatment of major depressive disorders (MDDs), act by inhibiting the reuptake of norepinephrine and serotonin and elevating the concentrations of these neurotransmitters within the synaptic cleft [33,34]. Amitriptyline belongs to the TCA class of antidepressants, and it has been FDA-approved to treat MDDs in adults [35] and used off-label to treat anxiety, post-traumatic stress disorder, insomnia, chronic pain (diabetic neuropathy, fibromyalgia), irritable bowel syndrome, interstitial cystitis (bladder pain syndrome), migraine prophylaxis, postherpetic neuralgia, sialorrhea [36,37,38] and post-COVID-19 headaches [39].

Recently, emerging evidence has suggested that several antidepressant drugs are able to interfere with autophagy, a highly conserved catabolic pathway that delivers cytoplasmic components to lysosomes for degradation [40], and this may be involved in their ability to suppress tumor growth [41,42,43,44,45].

The aims of the study were to elucidate whether exposure to amitriptyline (1) affected neuroblastoma cell survival and proliferation, (2) modulated the autophagy-lysosomal pathway and (3) if modulation of autophagy was responsible for the reported cytotoxic effects of the drug.

## 2. Results

### 2.1. Amitriptyline Induced a Concentration and Time-Dependent Reduction in Cell Viability in SH-SY5Y Cultures 

**Figure 1:**
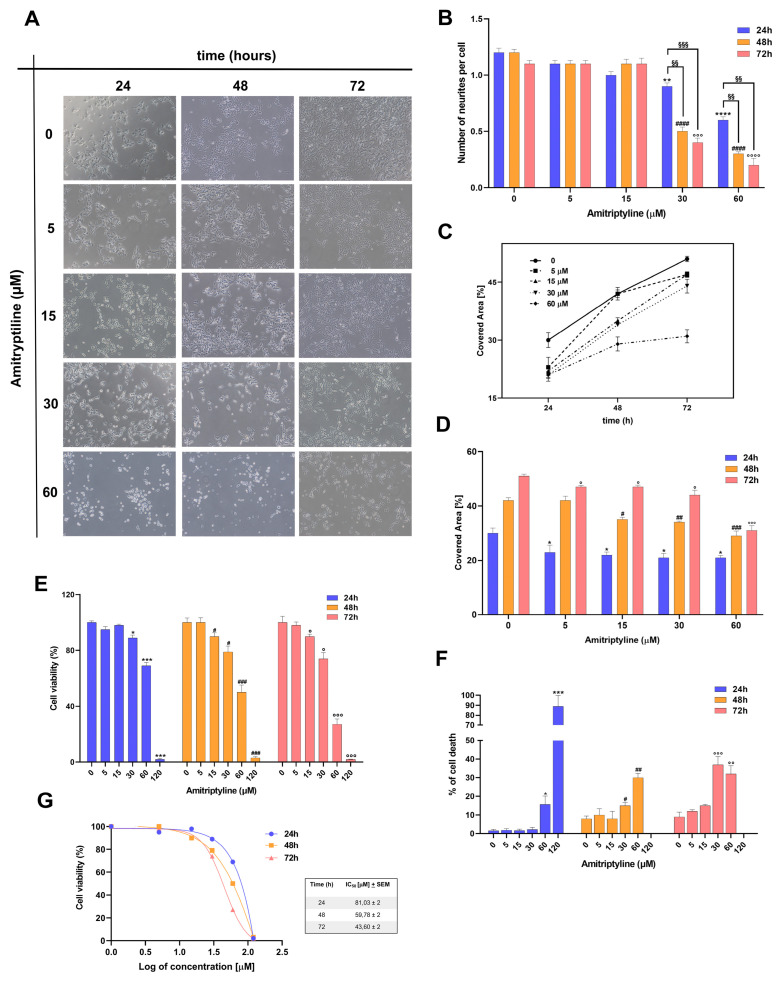
The effects of amitriptyline on cell morphology and viability of SH-SY5Y neuroblastoma cells. (**A**) Phase contrast microscopy images (10× objective) showing SH-SY5Y cell culture morphology under control conditions (0) and following treatment with amitriptyline 5–60 μM for 24, 48 and 72 h. (**B**–**D**) Histograms showing the number of neurites per cell (**B**) and the percentage of covered area (**C**) in SH-SY5Y control cultures (0) and cultures exposed to increasing concentrations of amitriptyline 5–60 μM for 24, 48 and 72 h. Each bar represents the mean ± s.e.m. (standard error of the mean) of 3–4 independent experiments. (**E**,**F**) The concentration-dependent cytotoxicity of amitriptyline in SH-SY5Y cell cultures. Cells were treated with amitriptyline 5–120 μM for 24, 48 and 72 h, and cell viability was evaluated by an MTT assay (**E**) or a trypan blue assay (**F**). Histograms represent the mean ± s.e.m. of 3–4 independent experiments. (**G**) IC_50_ was calculated for cell viability after 24, 48 and 72 h of exposure to amitriptyline. (* *p* < 0.05; ** *p* < 0.01; *** *p* < 0.001; **** *p* < 0.0001 vs. 0–24 h; # *p* < 0.05; ## *p* < 0.01; ### *p* < 0.001; #### *p* < 0.0001 vs. 0–48 h; ° *p* < 0.05; °° *p* < 0.01;°°° *p* < 0.001; °°°° *p* < 0.0001 vs. 0–72 h; §§ *p* < 0.01; §§§ *p* < 0.001).

Under control conditions, SH-SY5Y cells grow as monolayers displaying a flattened morphology and cytoplasmic extensions (Figure 1A). However, in cultures exposed to increasing concentrations of amitriptyline 15–60 μM for 24 h, a significant percentage of cells rounded up and a reduction in neuritic processes (Figure 1B) was observed in cells exposed to higher concentrations of 30–60 μM, suggesting that cell retraction and detachment occurred (Figure 1A–D). 

To evaluate whether these morphological changes were associated with or preceded cell death, cell viability was evaluated in SH-SY5Y exposed to amitriptyline for 24, 48 and 72 h. 

The treatment of cultures with amitriptyline 15–120 μM, but not with 5 μM, induced a concentration and time-dependent reduction in cell viability measured as mitochondrial activity (Figure 1E) and altered cell membrane integrity, evaluated by a trypan blue exclusion test (Figure 1F). 

IC_50_ was calculated for cell viability at each considered time point, and corresponded to 81.03 + 2, 59.78 + 2 and 43.60 + 2 μM at 24, 48 and 72 h, respectively (Figure 1G).

**Figure 2:**
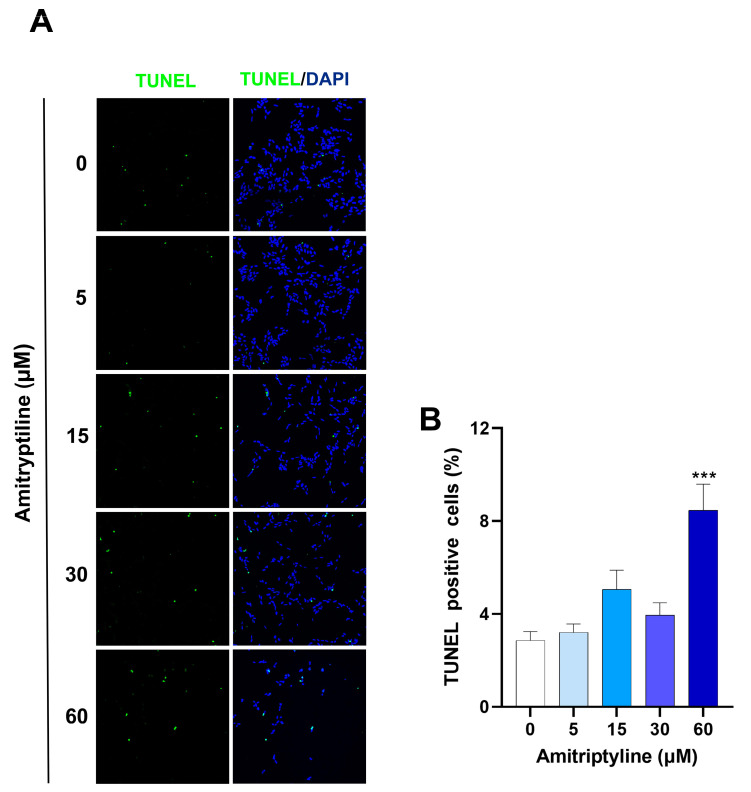
Analysis of TUNEL-positive cells in SH-SY5Y cultures exposed to amitriptyline for 24 h. (**A**) Representative microphotographs of cells treated with amitriptyline for 24 h and stained (green) by a TUNEL assay. Cell nuclei were counterstained with DAPI (blue). (**B**) The apoptotic cell index was calculated as the percentage of TUNEL-positive cells versus the total cells counted per microscopic field. A total of 10 fields were acquired for each experimental condition using a confocal microscope equipped with a 20× objective. Data were reported as the mean ± s.e.m. of n = 5 independent experiments. (*** *p* < 0.001 vs. control 0).

Since alteration of cell adhesion is often associated with apoptosis [46,47] we performed a TUNEL assay. As shown in Figure 2, the exposure of SH-SY5Y cells to amitriptyline 5, 15 or 30 μM for 24 h did not significantly increase the number of TUNEL-positive cells as compared to the control cultures, whereas a small but significant population of apoptotic cells was detected in cultures treated with amitriptyline 60 μM (Figure 2A,B). No activation of caspase-3 was observed by western blotting analysis at any of the concentrations and time points analyzed.

### 2.2. Amitriptyline Reduced SH-SY5Y Clonogenic Capacity

An analysis of the images obtained by phase-contrast microscopy showed that, at the seeded density, the surface area covered by growing cells exposed to amitriptyline 5–60 μM for 24, 48 and 72 h was significantly lower as compared to control cultures (Figure 1C,D). 

**Figure 3:**
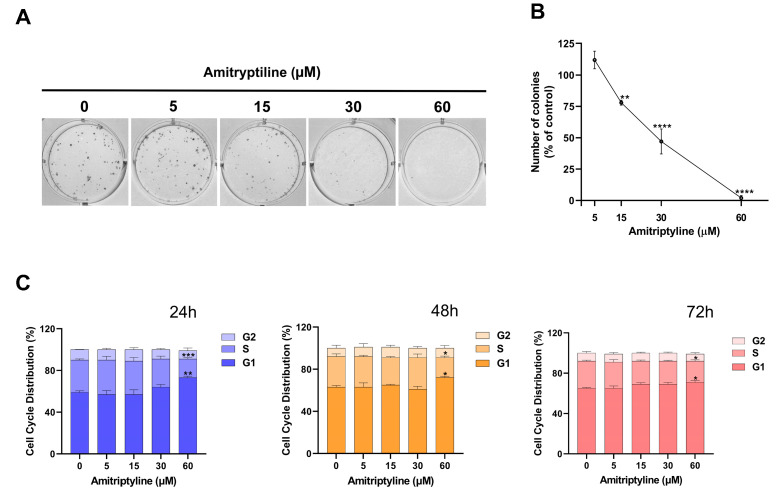
Amitriptyline reduces clonogenic capacity and affects the cell cycle distribution of SH-SY5Y cells. (**A**) A representative image of a clonogenic assay showing the concentration-dependent inhibition of colony formation induced by incubation with amitriptyline 15–60 μM for 12 days. (**B**) The quantification of the colonies formed expressed as the percentage of the untreated control (0). The results are reported as the mean ± s.e.m. of n = 5 independent experiments (** *p* < 0.01; **** *p* < 0.0001). (**C**) Histograms show the distribution of cell cycle phases following exposure to amitriptyline 5–60 μM for 24, 48 and 72 h. The results are expressed as the mean ± s.e.m. of n = 4 independent experiments (* *p* < 0.05; ** *p* < 0.01; *** *p* < 0.001 vs. untreated control 0).

This can be partially due to the cytotoxic effects of the treatment (Figure 1E,F). However, exposure to the tricyclic antidepressant may also alter the proliferation rate of neuroblastoma cells similar to what has been reported in other cell lines [25,48]. Therefore, we tested the effects of amitriptyline on the growth and proliferation of SH-SY5Y cultures. As shown in Figure 3, amitriptyline 15–60 μM significantly reduced the SH-SY5Y clonogenic capacity (i.e., the capability of a single cell to form a colony) in a concentration-dependent manner (Figure 3A,B). 

To further investigate the effects of amitriptyline on cell proliferation, we performed a cell cycle analysis in cultures exposed to increasing concentrations of amitriptyline (5–60 μM) for 24, 48 and 72 h (Figure 3C). Significant changes in cell cycle phase distribution, with an increase in S and G1 phases, were reported only when cultures were exposed to amitriptyline 60 μM. Lower concentrations of the antidepressant did not affect the distribution of cell cycle phases at any of the analyzed time points (5–30 μM) (Figure 3C). No significant changes were reported in the value of the hypodiploid SubG1 phase. 

### 2.3. Autophagy Is Modulated in SH-SY5Y Cell Cultures Exposed to Amitriptyline

It has been previously reported that tricyclic antidepressants affect the autophagy pathway in several immortalized and primary cells [41,49,50,51,52,53]. To investigate if the modulation of autophagy also occurs in SH-SY5Y cells exposed to amitriptyline, we studied the expression of the microtubule-associated protein 1 light-chain (LC3). LC3I is the soluble cytoplasmic form of the protein which, once converted in LC3II by lipidation, is stably associated with autophagosomes (i.e., the vesicular double membrane structures where the autophagic cargo is loaded) [54]. 

**Figure 4:**
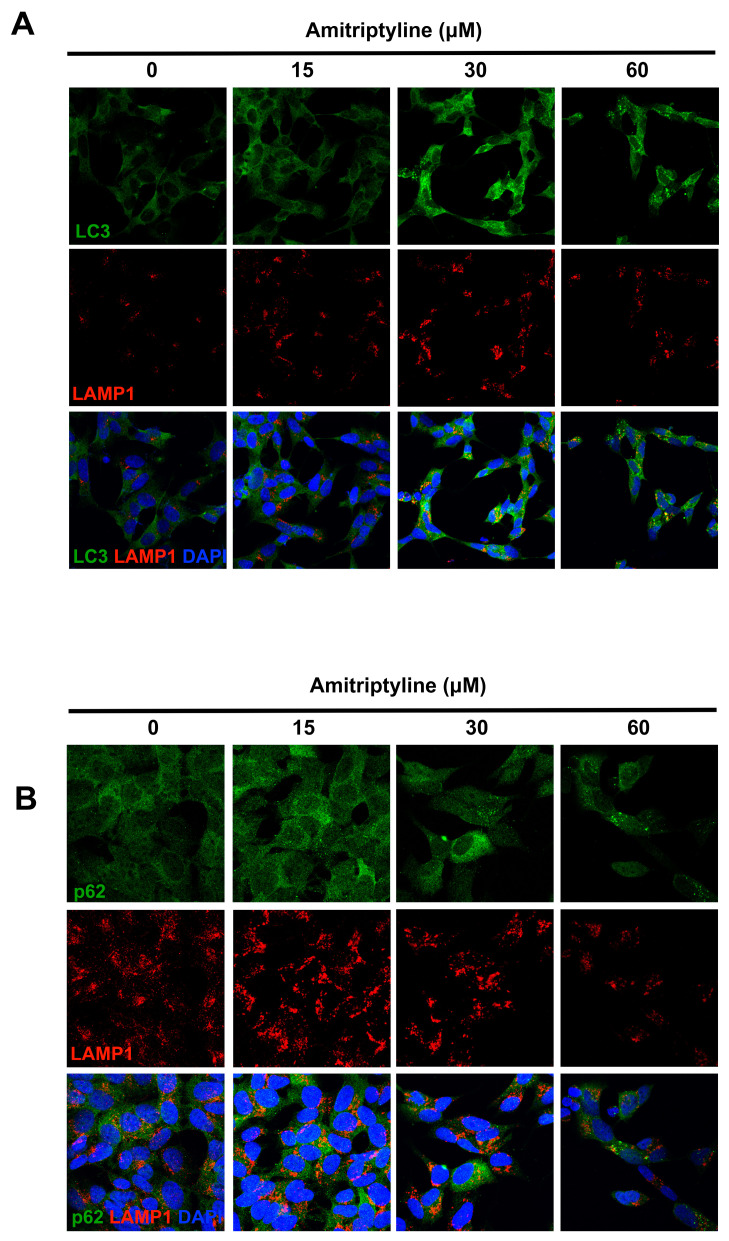
LC3, p62 and LAMP1 immunoreactivity in SH-SY5Y cells exposed to amitriptyline. (**A**) Representative confocal images of SH-SY5Y cells labeled with anti-LC3 (green) and anti-LAMP1 (red) antibodies. Following exposure to increasing concentrations of amitriptyline 15–60 μM for 24 h, LC3 immunoreactivity was upregulated in SH-SY5Y cultures as compared to control cultures and partially co-localized with LAMP1-positive structures. (**B**) Images showing the distribution of anti-p62 (green) and LAMP1 (red) immunoreactivity in neuroblastoma cells treated with amitriptyline 15–60 μM for 24 h. Nuclei were counterstained with DAPI (blue). (n = 4). Images were acquired with a confocal microscope equipped with a 20× objective with 2× zoom.

An immunofluorescence analysis showed that, following exposure to an increasing concentration of amitriptyline for 24 h, LC3 immunoreactivity was upregulated in SH-SY5Y cultures as compared to control cultures, with redistribution of the fluorescence signal in bright dots localized at the perinuclear region (Figure 4A). Most of these LC3-positive punctua colocalized with the lysosomal-associated membrane protein 1 (LAMP1), indicating that fusion of autophagosomes with lysosomes occurred (Figure 4A). 

**Figure 5:**
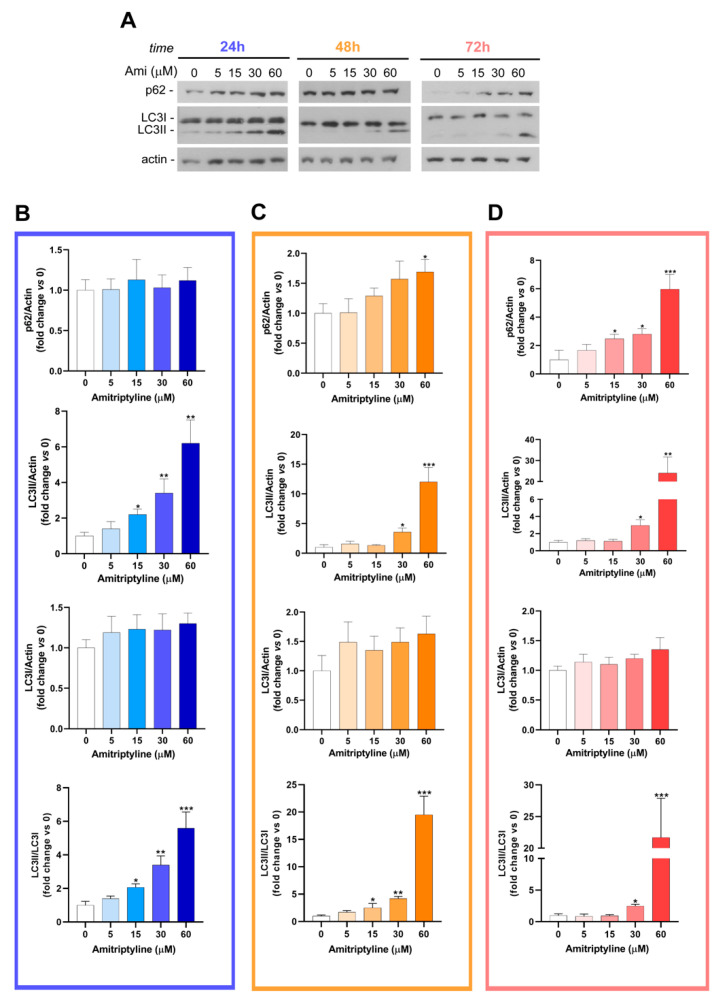
Amitriptyline modulates LC3 and p62 protein expression in SH-SY5Y neuroblastoma cells. (**A**) Representative immunoblot showing p62 and LC3 expression in SH-SY5Y cells exposed to amitriptyline 5–60 μM for 24 (**B**), 48 (**C**) and 72h (**D**). Actin was used as the loading control. (**B**–**D**) Histograms report the results of the densitometric analysis of the bands normalized to loading control following treatment with amitriptyline for 24, 48 and 72 h, respectively. Data are reported as the mean ± s.e.m. of n = 3–5 independent experiments. (Significance was determined via a Student’s *t* test; * *p* < 0.05, ** *p* < 0.01, *** *p* < 0.001 vs. untreated control 0).

Western blotting analysis confirmed that 24 h exposure to amitriptyline 15–60 μM significantly induced a concentration-dependent accumulation of LC3II and increased the LC3II/LC3I ratio (Figure 5A,B). This effect was maintained at 48 and 72 h but only when cultures were exposed to amitriptyline 30 or 60 μM (Figure 5A,C,D). The accumulation of LC3II was absent at lower concentrations of amitriptyline (5 μM) at all considered time points (Figure 5).

The upregulation of LC3II was associated, after 72 h incubation, with a concentration-dependent accumulation of the autophagy substrate and receptor p62/SQSTM-1, while no significant changes were reported at shorter time points (Figure 5). Nevertheless, 24 h of exposure to amitriptyline 15–60 μM led to a heterogeneous distribution of p62 immunoreactivity with the appearance of p62 bodies partially colocalizing with LAMP1-positive compartments (Figure 4B). 

This would suggest that, at this time point, p62/SQSTM-1 is efficiently recruited into the lysosomal compartment and, together with the upregulation of LC3II and its colocalization with LAMP1, this would indicate that the enhancement of functional autophagy is triggered by the TCA.

However, the delayed accumulation of p62 might imply that, after 72 h exposure, the efficiency of autophagosome clearance is impaired or insufficient in amitriptyline-treated cells. 

To confirm this hypothesis, we performed an autophagic flux assay by studying the effect of amitriptyline in the presence and absence of the lysosomal inhibitor BafA1, which prevents lysosomal acidification and blocks the fusion between autophagosomes and lysosomes [55]. 

**Figure 6:**
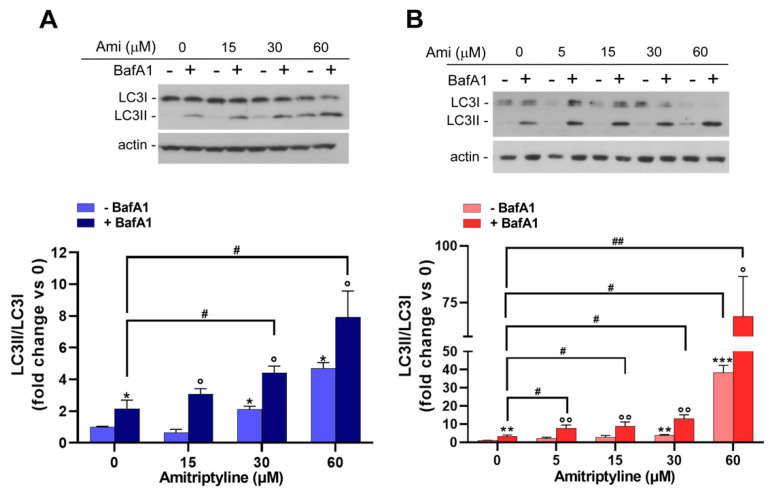
Amitriptyline increases autophagy flux in SH-SY5Y cells. A representative immunoblot showing the level of LC3 proteins in SH-SY5Y treated with amitriptyline for 24 (**A**) or 72 h (**B**) and incubated with the lysosomal inhibitor Bafilomycin A1 (+BafA1; 100 nM) or vehicle (−BafA1) for the last 4 h of treatment. Actin was used as the loading control. Histograms show the LC3II/LC3I ratio optical density ratio reported as the mean ± s.e.m. of n = 3–5 independent experiments. (The significance was determined via a Student’s *t* test; * *p* < 0.05; ** *p* < 0.01; *** *p* < 0.001 vs. 0 − BafA1; ° *p* < 0.05; °° *p* < 0.01, vs. the corresponding concentration of amitriptyline −BafA1; # *p* < 0.05; ## *p* < 0.01).

When lysosomal activity was inhibited by BafA1, a significant increase in the LC3II/LC3I ratio was still reported in cultures exposed to amitriptyline for 24 (Figure 6A) or 72 h (Figure 6B) as compared to vehicle-treated cells supporting, the hypothesis that the rate of autophagosomal formation was enhanced by the antidepressant. 

### 2.4. Amitriptyline Does Not Affect Lysosomal pH but Induces Lysosomes Accumulation

Lysosomes are highly dynamic organelles serving as degradation hubs for autophagy. Based on its cationic amphiphilic properties, the off-target effects of amitriptyline could be linked to its accumulation within lysosomes [56,57,58]. 

**Figure 7:**
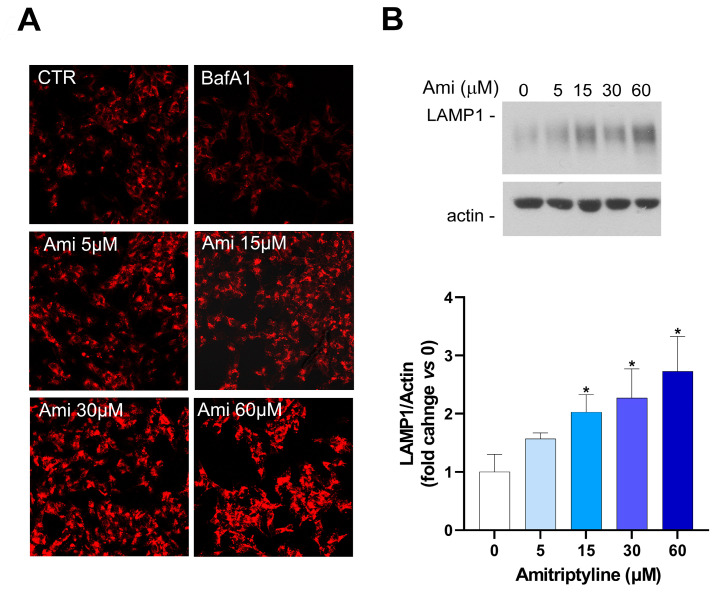
The effects of amitriptyline on lysosomal pH and LAMP1 expression in SH-SY5Y cells. (**A**) Representative images of Lysotracker Red (LTR) staining of SH-SY5Y cells exposed to amitriptyline 5–60 μM for 24 h. BafA1 (100 nM) was used as the positive control to reduce the lysosomal pH. A concentration-dependent increase in LTR intensity is reported after 24 h exposure to AMI. Images were acquired with a confocal microscope equipped with a 20× objective. (**B**) Western blotting analysis showing the dose-dependent increase in LAMP1 expression in SH-SY5Y cells treated with amitriptyline 5–60 μM for 24 h. Histograms show the densitometric analysis of the bands normalized to the loading control (actin) and are reported as the mean ± s.e.m. of n = 3 independent experiments. (The significance was determined via Student’s *t* test; * *p* < 0.05).

To investigate the effects of amitriptyline on the lysosomal arm of the autophagy pathway, we used the fluorescent dye Lysotracker Red (LTR), an acidotropic probe that is trapped and therefore labels cellular acidic compartments, including lysosomes and autolysosomes [59]. As expected, BafA1, which acts as a specific inhibitor of vacuolar-type H+-ATPase [60] and was here used as the positive control, reduced LTR intensity compared to the control cultures (Figure 7A). On the contrary, a concentration-dependent increase in LTR intensity was reported after 24 h of exposure to amitriptyline, suggesting that no changes in acidic lysosomal pH occurred at any of the tested antidepressant concentrations (Figure 7A).

Staining with the fluorescent dye also showed a concentration-dependent increase in the cytoplasmic area occupied by acidic compartments in cells exposed to amitriptyline 5–60 μM for 24 h (Figure 7A). 

This observation was supported by LAMP1 western blotting analyses. Indeed, a concentration-dependent increase in LAMP1 expression was detected after 24 h of treatment with the antidepressant at the concentration of 15–60 μ (Figure 7B).

### 2.5. Autophagy Modulation Does Not Take Part to the Cytotoxic Effects of Amitriptyline

To investigate if the modulation of autophagy was involved in the reduction in cell viability and clonogenic capacity induced by amitriptyline, cells were preincubated with chloroquine (CQ), an autophagy inhibitor that prevents autophagosome–lysosome fusion and blocks the degradative activity of lysosomes [60]. 

**Figure 8:**
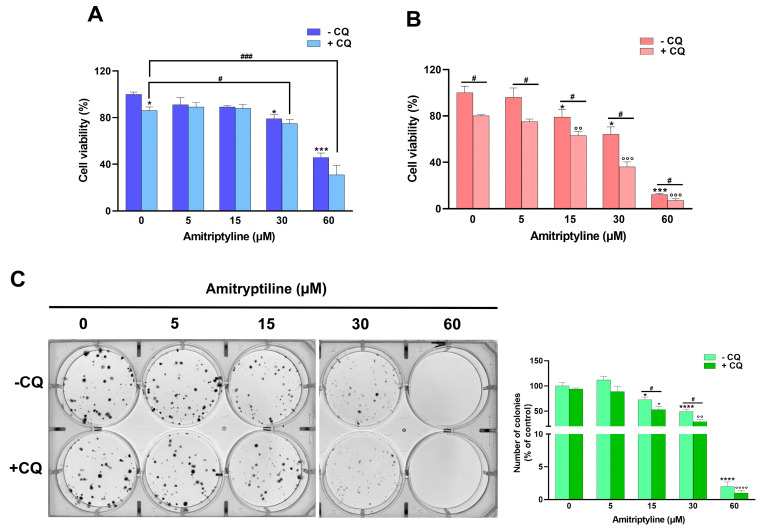
The inhibition of autophagy by chloroquine does not revert the cytotoxic and antiproliferative effects of amitriptyline in SH-SY5Y cells. SH-SY5Y cells were preincubated with chloroquine (CQ, 20 μM) for 2 h and then exposed to amitriptyline 5–60 μM for 24 (**A**) and 72 h (**B**). Cell viability was evaluated by an MTT assay. CQ itself significantly reduced cell viability in untreated cells and exacerbated the cytotoxic effect of amitriptyline after 72 h incubation. Data (the mean ± s.e.m. of n = 5 independent experiments) are expressed as the percentage of the untreated control (0 − CQ). (**C**) Representative image of clonogenic assay of SH-SY5Y pretreated with CQ and incubated with amitriptyline 5–60 μM for 12 days. The graph shows the quantification of the colonies formed in four independent experiments; the results (the mean ± s.e.m.) are expressed as relative colony numbers compared to untreated cells (0 − CQ). (* *p* < 0.05; *** *p* < 0.001; **** *p* < 0.0001 vs. 0 − CQ; ° *p* < 0.05; °° *p* < 0.01; °°° *p* < 0.001; °°°° *p* < 0.0001 vs. corresponding concentration of amitriptyline − CQ; # *p* < 0.05; ### *p* < 0.001).

As shown in Figure 8, CQ itself significantly reduced cell viability in control cultures and further potentiated the effect of amitriptyline after 72 h of incubation (Figure 8B). No significant effects due to autophagy inhibition were reported in cultures exposed to amitriptyline 5–30 μM for 24h, while a further reduction in cell viability was detected at the highest concentration tested (60 μM) (Figure 8A). 

**Figure 9:**
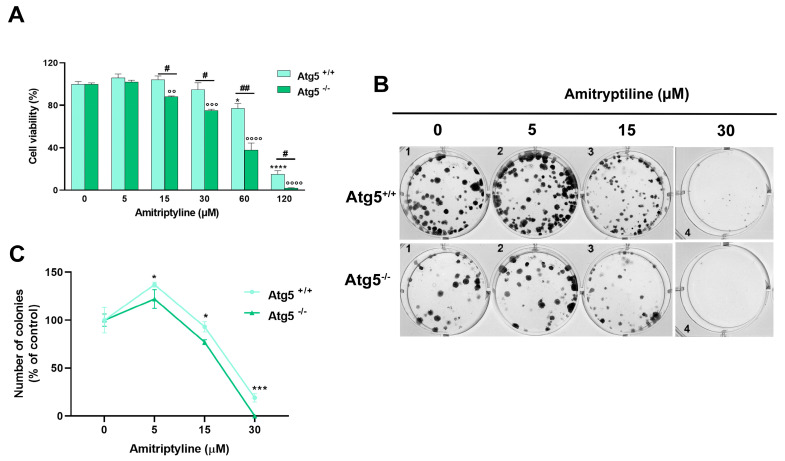
The effects of amitriptyline on cell viability and clonogenic capacity in autophagy-deficient Atg5^−/−^ mouse embryonic fibroblast (MEF) (**A**) Atg5-deficient (Atg5^−/−^) and wild-type (Atg5^+/+^) mouse embryonic fibroblasts (MEFs) were treated with amitriptyline 5–120 μM for 24 h and cell viability was measured by an MTT assay. Cell viability was significantly reduced in Atg5^−/−^ compared to Atg5^+/+^ MEF following treatment with amitriptyline 15–120 μM. Histograms represent the mean ± s.e.m. of 3 independent experiments (* *p* < 0.05; **** *p* < 0.0001 vs. 0 Atg5^+/+^; °° *p* < 0.01; °°° *p* < 0.001; °°°° *p* < 0.0001 vs. 0 Atg5^−/−^; # *p* < 0.05; ## *p* < 0.01). (**B**) Representative images of a clonogenic assay in Atg5^−/−^ and Atg5^+/+^ MEF cells treated with amitriptyline. (**C**) The quantification of the colonies expressed as the percentage of the relative untreated control (0). Results are reported as the mean ± s.e.m. of n = 4–5 independent experiments (* *p* <0.05; *** *p* < 0.001 vs. the relative untreated control).

To strengthen the observation that the modulation of autophagy is not the key mechanism responsible for the cytotoxic effect of amitriptyline but rather a cytoprotective pathway triggered by exposure to the antidepressant, we evaluated the effect of amitriptyline in autophagy-deficient Atg5^−/−^ MEF. As reported in Figure 9A, autophagy deficiency drastically reduced cell viability in cells exposed to amitriptyline 15–120 μM compared to wild-type Atg5^+/+^ MEF. No effect on viability was reported in both autophagy-deficient and wild-type MEF treated with a lower concentration of amitriptyline (5 μM) (Figure 9A).

### 2.6. Reduced Clonogenic Capacity in Amitriptyline Treated Cells Does Not Depend on Autophagy Modulation

To investigate the relation between the modulation of autophagy and the reduced clonogenic capacity in amitriptyline-treated neuroblastoma cells, we used the autophagy-deficient MEF. However, as shown in Figure 9B, the deletion of the Atg5 gene in the Atg5^−/−^ MEF significantly altered the morphology and distribution of colonies as compared to Atg5^+/+^ MEF, making the results following the treatment not comparable (Figure 9B,C).

Then, the capacity of amitriptyline to suppress colony formation was evaluated in SH-SY5Y pretreated with the autophagy inhibitor CQ. The inhibition of autophagy significantly reduced the number of colonies formed after 12 days of exposure to amitriptyline 15 and 30 μM (Figure 8C), suggesting that autophagy does not cause, but rather buffers, the effect of amitriptyline on cell proliferation.

## 3. Discussion

Autophagy is an evolutionary, conserved catabolic pathway that ensures organelle and protein homeostasis through their lysosomal degradation [40]. This process represents a critical cellular response to either physiological and pathological stimuli and its alteration, or an inherited mutation on autophagy-related genes (Atg), and has been linked to several human diseases, including neurodegenerative diseases and cancer [61,62,63,64]. Several common drugs have been found to be able to modulate autophagy with molecular mechanisms that have been only partially identified and are often independent of their main pharmacological targets [65,66,67]. This implies the opportunity to repurpose those drugs in diseases with known alterations of the autophagy pathway and, on the other hand, raises questions regarding the possibility that some therapeutic treatments may affect the progression of co-existing diseases in which autophagy plays a relevant role.

In the present study, we showed that in SH-SY5Y neuroblastoma cells, amitriptyline, a TCA drug prescribed for depressive syndromes and pain, modulates autophagy in a time and dose-dependent manner with the upregulation of the autophagosome-associated form of LC3, LC3II, and a delayed accumulation of the autophagy receptor/substrate p62. The effects on autophagy were not involved in the decreased cell viability and clonogenic capacity observed following exposure of neuroblastoma cultures to the TCA, since the genetic and pharmacological inhibition of autophagy did not prevent, but rather increased, amitriptyline cytotoxicity.

Recent studies have demonstrated that diverse antidepressant drugs have inherited anticancer activity; in particular, TCAs are cytotoxic to several cancer cell lines in vitro [68,69,70] and are able to reverse multidrug resistance of tumor cells in vitro and in tumor-bearing mice [50,71,72,73].

Amitriptyline has been shown to exert, through different molecular mechanisms, antitumor effects in several types of cancers, including colon carcinoma, lung cancer, breast cancer, glioblastoma, multiple myeloma, melanoma and hepatocarcinoma [74,75,76,77].

Apoptotic cell death was induced by amitriptyline in human multiple myeloma cell lines and primary cells by decreasing histone deacetylase (HDAC) (HDAC-3, 6, 7, 8) expression and inhibiting HDAC activity; furthermore, amitriptyline reduced cyclin D2 expression arresting cells in the G0/G1 phase of the cell cycle [78]. Cell viability and proliferation of uterine leiomyosarcoma cells were suppressed by amitriptyline treatment, and apoptosis induction was mediated by the upregulation of the non-selective neurotrophin receptor (NTR) p75NTR [79]. In lung cancer cells, amitriptyline activated TRAIL (tumor necrosis factor-related apoptosis-inducing ligand)-induced apoptosis by increasing death receptors (DR) 4 and 5 [80]. Decreased tumor cell proliferation through a reduction in Ki-67 and the inhibition of β-catenin [81] was reported in hepatocellular carcinoma cells treated with amitriptyline, while in glioblastoma multiforme (GBM), amitriptyline induced cell death by interfering with mitochondrial function [82].

Here, we observed a dose and time-dependent cytotoxic effect of amitriptyline in human neuroblastoma cells, reporting a reduction n cell viability and cell proliferation. However, under our experimental conditions, we did not detect a direct and quantitively reasonable correlation with apoptotic hallmarks, such as DNA fragmentation, caspase-3 activation and an increase in hypodiploid events. Therefore, it can be hypothesized that other forms of cell death are activated in neuroblastoma cells following exposure to the TCA.

This is supported by the work of Lee and colleagues which reported that, in SH-SY5Y cells, amitriptyline and desipramine induced cell death, but not apoptosis [83]. Indeed, cell death, as well as mitochondrial damage and oxidative stress induced by the TCA, was attenuated by antioxidants but not by inhibitors of caspases, Parp-1, cathepsin or calpains, suggesting that it was different from conventional apoptosis or programmed necrosis [83]. Accordingly, caspase-independent cell death following exposure to amitriptyline has been reported in hepatoma HepG2 cells [84]. In this same study, an early activation of autophagy, with an increase in LC3II, Beclin-1 and Atg12-Atg5, and the upregulation of LAMP1, was reported, suggesting that autophagy activation preceded cell death that eventually occurred by necrosis or autophagy–apoptosis switch [84,85].

In our study, we observed an early increase in LC3 lipidation which occurs in the first 24 h of exposure to amitriptyline. Autophagy flux experiments showed that amitriptyline is still able to increase LC3II levels even under the presence of the autophagy inhibitor BafA1, implying that treatment with the TCA is associated with autophagy induction rather than the inhibition of autophagosomal degradation. This hypothesis is also supported by the increase in LC3 immunofluorescence signals in cells treated with amitriptyline and the colocalization observed between LC3/p62 and p62/LAMP1. However, the delayed accumulation of the autophagy substrate p62 would suggest that the formation of autophagosomes overcomes the cell capacity to degrade their content through the lysosomal system, therefore limiting autophagosomal turnover.

Basic lipophilic compounds, like amitriptyline and other TCAs, accumulate into acidic intracellular compartments, such as lysosomes, and may perturb the vesicular pH, therefore disturbing the autophagy process [49,86,87,88,89]. However, in our study, the acidic lysosomal pH was not modified by treatment with amitriptyline, as demonstrated by the efficient loading of the acidotropic probe LTR. In agreement with this observation, a study by Kornhuber and colleagues showed that the lysosomal pH is not changed by amitriptyline [90]. Nevertheless, the accumulation of amitriptyline into the lysosomal lumen, as a lysosomotropic drug, may affect lysosomal membrane permeability [90] and lysosomal enzyme activity, accounting for the delay of autophagic cargo degradation [91,92].

Several studies on the cytotoxic effects of TCAs have shown that these is often mediated by autophagy dysregulation [48]. However, controversial findings on the influence of amitriptyline on autophagy and its role in the cytotoxicity of antidepressants among different types of cancer have been reported. For example, in hepatocellular carcinoma cells, amitriptyline induces an early autophagy activation, and pharmacological or genetic inhibition of autophagy exacerbates the toxic effects of amitriptyline, thus increasing apoptosis [84]. Vice versa, in lung cancer cells, amitriptyline inhibited autophagy by blocking the fusion of autophagosomes with lysosomes; the amitriptyline-induced autophagy blockade increased DR4 and DR5 expression, enhancing TRAIL-mediated apoptotic cell death [80].

In our study, the inhibition of autophagy by CQ, which prevents the process of autophagosome–lysosome fusion, did not exert significant effects on cell viability when cultures were exposed to amitriptyline for 24 h, while the cytotoxic effects of the antidepressant were exacerbated after longer exposure and further reduced clonogenic capacity. These data support the hypothesis that, under our experimental conditions, the modulation of autophagy is activated by amitriptyline as a cytoprotective mechanism; however, the newly formed autophagosomes may not be efficiently degraded through the lysosomal system, either because it is overloaded and above the degradation rate or functionally impaired. This hypothesis is also supported by previous observations, showing that when autophagy is inhibited in the step of autophagy induction (i.e., 3-MA treatment), TCA-induced cell death is not reduced neither aggravated [83] since it occurs before the overloading of the lysosomal degradation system.

Although our data demonstrate that autophagy and cytotoxicity are two uncorrelated events occurring in neuroblastoma cells exposed to amitriptyline, we must highlight the limitation of this study that stem mainly from the use of a single cell line and the still unidentified molecular mechanisms underlaying the observed effects. In view of this, further studies are guaranteed, aiming at extending our observations in other cellular systems and dissecting the pathways, beside autophagy, involved in the antiproliferative and cytotoxic effects of amitriptyline.

Several tasks still need to be completed to rationalize the data in terms of clinical applications.

## 4. Conclusions

In summary, our study shows that amitriptyline exerts cytotoxic and antiproliferative effects on neuroblastoma cells while modulating autophagy. The induction of TCA-mediated autophagy is a protective mechanism, and it is not responsible for the observed cytotoxicity. Further studies are needed to investigate the molecular mechanisms underlying the reported effects and to translate these observations in vivo. Nevertheless, our study, together with the previously published literature, poses the basis for further investigation on the potential exploitation of these effects for therapeutic interventions and on the consequences of long-term antidepressant treatment.

## 5. Materials and Methods

### 5.1. Reagents

Amitriptyline hydrochloride (A8404), dimethyl sulfoxide (DMSO), chloroquine (CQ; C6628) and bafilomycin A1 (BafA1; B1793) were purchased from Sigma-Aldrich (Milan, Italy).

### 5.2. Cell Cultures and Treatments

Adherent human SH-SY5Y neuroblastoma cells, obtained from ICLC-IST (Genoa, Italy), were grown in an RPMI 1640 medium (Gibco, Life Technologies, Paisley, UK) supplemented with 10% heat-inactivated fetal bovine serum (FBS; Gibco, Life Technologies, Paisley, UK), 1 mM sodium pyruvate, 2 mM glutamine (Gibco, Life Technologies, Paisley, UK), 100 IU/mL penicillin and 100 μg/mL streptomycin (Gibco, Life Technologies, Paisley, UK).

Wild-type (Atg5^+/+^) and Atg5-deficient (Atg5^−/−^) mouse embryonic fibroblasts (MEFs) were purchased from RIKEN Bio-Resource Cell Bank (Japan) and grown in Dulbecco’s modified Eagle’s medium (DMEM, Gibco, Life Technologies, Paisley, UK) supplemented with 10% FBS, 100 IU/mL penicillin (Gibco, Life Technologies, Paisley, UK) and 100 μg/mL streptomycin (Gibco, Life Technologies, Paisley, UK). Cell cultures were maintained at 37 °C in a humidified atmosphere with 5% CO_2_.

Cells, cultured in 75 cm^2^ flasks, were seeded onto 96, 24 or 6-well plates; 24 h after plating, the medium was replaced with a fresh medium (control), a medium supplemented with a vehicle or the indicated compound. A stock solution of amitriptyline (5 mM) was prepared in water and diluted in a culture medium to obtain the final concentrations of 5–120 μM. Amitriptyline concentrations were chosen based on previously published in vitro studies [49,83].

CQ, dissolved in water (50 mM) and diluted in a culture medium, was applied to SH-SY5Y cells at a final concentration of 50 μM [93], 2 h before the addition of the antidepressant, and was maintained throughout the period of treatment.

A stock solution of BafA1 (1 mM) was prepared in DMSO and further diluted in a culture medium to the final concentration of 100 nM [94]; cells were treated with BafA1 for the last 4h of exposure to amitriptyline. DMSO (0.01%) was added to the medium of vehicle-treated cultures.

### 5.3. Cell Viability Study

Cell viability was assessed by a trypan blue dye exclusion test (0.4% *w/v*) and cell death was reported as the percentage of stained (non-viable) cells vs. total cells counted [95].

Cell metabolic activity, as indirect measure of cell viability, was evaluated by the quantification of the intracellular reduction of the yellow dye 3-[4,5-dimethylthiazol-2-yl]-2,5 diphenyl tetrazolium bromide (MTT; Sigma-Aldrich, Milan, Italy) to purple formazan. Cells, seeded and treated onto 96-well plates, were incubated with 100 μL of MTT (0.5 mg/mL) for 1h in a humidified 5% CO_2_ incubator at 37 °C. At the indicated time points (24, 48 or 72 h) the medium was removed, and formazan crystals were solubilized by the addition of 100 μL DMSO. The absorbance was measured at 540/690 nm by a microplate spectrophotometer (Synergy H1 plate reader, BioTek Instruments, Inc., Winooski, VT, USA). For each experimental condition, the absorbance of 4–8 wells was averaged. Data were expressed as the percentage of cell survival vs. the control cultures (set to 100%). Each experiment was performed in triplicate.

### 5.4. Immunocytochemistry

For immunocytochemical staining, SH-SY5Y cells were plated onto poly-L-lysine (Sigma-Aldrich, Milan, Italy)-coated coverslips and cultured for 24 h before being exposed to amitriptyline 5–60 μM. At the indicated time, the culture medium was removed and the cells were washed with phosphate-buffered saline (PBS, pH 7.4), fixed with formalin solution (containing 4% paraformaldehyde; Sigma-Aldrich, Milan, Italy) for 15 min at room temperature (RT) and washed three times in PBS. Cells were permeabilized with a solution of Triton 0.1% for 10 min at RT, washed three times with PBS and blocked with 10% donkey serum (DS; Sigma-Aldrich, Milan, Italy) for 30 min. Coverslips were incubated with primary antibodies against rabbit microtubule-associated protein 1 light chain 3 (LC3; code PD036, MBL International Corporation, Nagoya, Japan; 1:500 dilution), mouse lysosomal-associated membrane protein 1 (LAMP1; Developmental Studies Hybridoma Bank, Iowa City, IA, USA; 1:200 dilution) and rabbit sequestosome 1 (p62/SQSTM1; code sc-25575, Santa-Cruz Biotechnology, Dallas, TX, USA; 1:50 dilution) in 5% DS/PBS overnight at 4 °C. After three washes with PBS, coverslips were incubated with Alexa Fluor secondary antibodies (Alexa Fluor 488 donkey anti-rabbit (1:500 dilution) and Alexa Fluor 594 donkey anti-mouse (1:1000 dilution) (Molecular Probes, Life Technologies Paisley, UK) for 1h at RT. Control experiments were prepared in the absence of a primary antibody to exclude secondary antibody non-specific staining. Coverslips were mounted with a Vectashield solution containing 4′,6-diamidino-2-phenylindole (DAPI, AB104139, Abcam, Cambridge, UK) to visualize the nuclei. Images were acquired using a confocal microscope (FV3000, Olympus Corporation, Tokyo, Japan).

### 5.5. Phase-Contrast Microscopy, Measurement of Cells Confluence and Neurites Count

SH-SY5Y cells were observed under an inverted phase contrast microscope (CKX53, Olympus Corporation, Tokyo, Japan) with a 10× objective, and 4 images for each condition were taken by an investigator blinded to the treatment and analyzed with the ImageJ software version 1.53k. The percentage value of the white area (cells) divided by the total area of the image corresponded to the value of confluence indicated as the percentage of the area covered by cells [96].

The number of neurites for each cell was quantified using the ImageJ software package (NIH, Bethesda, MD, USA) [97]. Thirty cells for each experimental condition were analyzed by a blinded investigator. Data were expressed as the average of the neurite number/cell. Each experiment was performed in triplicate.

### 5.6. Live-Cell Labeling of Acidic Compartments

For lysosomal staining, SH-SY5Y cells were grown on glass-bottom dish and treated with amitriptyline (5–60 μM) for 24 h. Cells were washed with media and loaded with a lysosomal probe (LysoTracker Red DND-99, Thermo Fisher Scientific, Waltham, MA, USA). LysoTracker (60 nM) was used according to the manufacturer’s instruction. Images were acquired by a confocal microscope (FV3000; Olympus Corporation, Tokyo, Japan).

### 5.7. TUNEL Assay

Apoptosis was detected using the DeadEnd™ Fluorometric TUNEL (Terminal deoxynucleotidyl transferase dUTP nick end labelling) system (cod. G3250, Promega Madison, Wisconsin, USA) according to the manufacturer’s instructions. Briefly, SH-SY5Y cells, cultured on coverslips, were fixed with 10% PFA, rinsed three times with PBS, and equilibrated with 100 µL Equilibration Buffer at RT for 10 min. Fixed cells were incubated with 50 µL of a Terminal Deoxynucleotidyl Transferase (TdT) solution for 1h at 37 °C in a humidified environment. The reaction was stopped by incubation with 2× SSC solution for 15 min and the coverslips were mounted with the Vectashield mounting media with DAPI (Abcam, Cambridge, UK) to visualize the nuclei. Images were acquired using a confocal microscope (FV3000, Olympus Corporation, Tokyo, Japan). TUNEL-positive cells were counted by an investigator blinded to the treatment and were expressed as the ratio of TUNEL-positive cells/total cells.

### 5.8. Protein Extraction and Western Blot Analysis

Cells were lysed in an ice-cold RIPA buffer (50 mM Tris-HCl, pH 8, 150 mM NaCl, 1 mM EDTA, 0.1% SDS, 1% IGEPAL and 0.5% sodium deoxycholate) containing protease inhibitor (code P8349; Sigma-Aldrich, Milan, Italy) and phosphatase inhibitor cocktails (code 05726; Sigma-Aldrich, Milan, Italy). Lysates were centrifuged for 15 min at 14,000× g at 4 °C, and supernatants were assayed for protein content with a Bio-Rad DC Protein Assay Kit (Bio-Rad Laboratories, Milan, Italy). An equal amount (10 μg) of total proteins for each condition was resolved by 8, 12 or 15% SDS-polyacrylamide gel electrophoresis (PAGE) and transferred onto PVDF membranes (Immobilon-P, Sigma-Aldrich, Milan, Italy). Membranes were blocked with 5% non-fat milk (Santa Cruz Biotechnology, Dallas, TX, USA) in Tris-buffered saline (TBS) containing 0.05% Tween 20 for 1 h at RT. The primary antibodies were incubated overnight at 4 °C, and then with a horseradish peroxidase (HRP)-conjugated secondary antibody for 1 h at RT. Protein bands were visualized with a ECL Western Blotting Detection kit (Santa Cruz Biotechnology, Dallas, TX, USA) and the chemiluminescence signal detected using X-ray films (Santa Cruz Biotechnology, Dallas, TX, USA). Autoradiographic films were scanned, digitalized at 600 dpi and band quantification was performed using ImageJ software (NIH, Bethesda, MD, USA).

The following primary antibodies and dilutions were used: anti-LC3 1:2000 (code PD036; MBL International Corporation, Nagoya, Japan; 1:500 dilution), anti-p62/SQSTM1 1:4000 (code P0067, Sigma-Aldrich, Milan, Italy), anti-actin 1:1000 (clone AC-40; Sigma-Aldrich, Milan, Italy) and anti-LAMP1 1:1000 (Developmental Studies Hybridoma Bank, Iowa City, IA, USA). HRP-conjugated goat anti-rabbit or anti-mouse IgG (Pierce Biotechnology, Rockford, IL, USA) were used as the secondary antibodies.

### 5.9. Clonogenic Assay

SH-SY5Y, Atg5^+/+^ and Atg5^−/−^ MEF cells, plated into 6-well plates at a density of 500 and 300 cells/well, respectively, were treated with a medium containing amitriptyline (5–120 μM) for 12 days. Cells were fixed and stained for 30 min with a mixture of 6.0% glutaraldehyde and 0.5% crystal violet (Sigma-Aldrich, Milan, Italy). Plates were imaged using an inverted phase contrast microscope (CKX53; Olympus Corporation, Tokyo, Japan) and colonies with more than 50 cells were automatically counted using the “colony blob count tool” of the NIH ImageJ software (Bethesda, MD, USA) by a blind investigator.

### 5.10. Cell Cycle Analysis

SH-SY5Y cells were plated into 6-well plates and 24 h later treated with amitriptyline (5–60 μM) for 24, 48 or 72 h. Cells were washed twice with PBS and fixed with ice-cold 70% ethanol overnight at −20 °C. After centrifugation, a solution containing 20 μg/mL propidium iodide (PI), 0.17 mg/mL RNAse-A and 0.1% Triton X-100 (Sigma Aldrich, Milan, Italy) was added to each tube and cells were stained for 20 min at RT in the dark and under constant agitation. The DNA content was measured by flow cytometry (CytoFLEX Beckman, Beckman Coulter, Milan, Italy). Data analysis was performed using CytExpert Beckman Coulter software version 2.4 (Beckman Coulter, Milan, Italy).

### 5.11. Statistical Analysis

Data are expressed as the mean ± S.E.M. of the indicated number of independent experiments and evaluated statistically for differences by an ANOVA followed by a Tukey–Kramer test for multiple comparisons. Where indicated, a Student’s *t* test was used to evaluate differences between the two means. A value of *p* < 0.05 was considered statistically significant. The statistical significance was analyzed using the GraphPad Prism 8.3.0 software (GraphPad Soft-ware, Inc., San Diego, CA, USA).

## Data Availability

Dataset available on request from the authors.
